# The effect of the melatonin on cryopreserved mouse testicular cells

**Published:** 2016-01

**Authors:** Ghasem Saki, Mehri Mirhoseini, Masoud Hemadi, Ali Khodadadi, Fereshteh Beygom Talebpour Amiri

**Affiliations:** 1 *Cellular and Molecular research Center, School of Medicine. Ahvaz Jundishapur University of Medical Sciences, Ahvaz, Iran.*; 2 *Faculty of Nursing and Midwifery, Mazandaran University of Medical Sciences, Sari, Iran.*; 3 *Infertility and Perinatology Research Center, School of Medicine, Ahvaz Jundishapur University of Medical Sciences, Ahvaz, Iran.*; 4 *Petroleum and Environmental Research Center, School of Medicine, Ahvaz Jundishapur University of Medical Sciences, Ahvaz, Iran.*; 5 *Department of Anatomy, Faculty of Medicine, Mazandaran University of Medical Sciences, Sari, Iran*

**Keywords:** *Mouse*, *Testicular cells*, *Melatonin*, *Apoptosis*

## Abstract

**Background:**

After improvements in various cancer treatments, life expectancy has been raised, but success in treatment causes loss of fertility in many of the survived young men. Cryopreservation of immature testicular tissues or cells introduced as the only way to preserve fertility. However, freezing has some harmful effects. Melatonin, a pineal gland hormone, has receptors in reproductive systems of different species. It is assumed that melatonin has free radical scavenger properties.

**Objective:**

The aim of this study was to evaluate the effects of melatonin on the cryopreserved testicular cells in mouse.

**Materials and Methods:**

Cells from 7- 10 days old NMRI mice testes were isolated using two step enzymatic digestion. The testicular cells were divided into two groups randomly and cryopreserved in two different freezing media with and without the addition of 100 µm melatonin. Finally, apoptosis of the cells was assayed by flow cytometry. Also, lactate dehydrogenase activity test was performed to assess the cytotoxicity.

**Results:**

The results of lactate dehydrogenase showed the nearly cytotoxic effect of melatonin. The results of flow cytometry showed increase in apoptosis in the cryopreserved cells in the media containing melatonin compared to the control group.

**Conclusion:**

The present study shows that melatonin has an apoptotic effect on cryopreserved mouse testicular cells.

## Introduction

There have been extensive advances in the treatment of childhood cancer. As a result, life expectancy has been raised, up to 80% of children survive their diseases; and the population of long-term adult survivors of childhood malignancies is growing (1-3). Unfortunately, these treatments may be toxic to the gonads (4). Semen cryopreservation is a well-developed technique, which is routinely used in many infertility laboratories (5). Cryopreservation is currently (and probably) the best method for long term preservation of spermatogonial stem cells (6). 

However, for prepubertal boys who have to undergo chemotherapy or radiotherapy for cancer and cannot produce an ejaculation containing spermatozoa, so an optimized method of testicular tissue cryopreservation is needed (5). There are three different ways for cryobanking of immature testicular tissue containing testicular cell suspension, testicular pieces and entire gonad (7). Testicular stem cell cryopreservation appears the only way to preserve fertility in pre-pubertal testicular cancer patients. Efficacious testicular stem cell transplantation, fresh or frozen-thawed suspension forms has been reported in the recent years (8). A method is developed specifically for cryopreservation of type a spermatogonia, including spermatogonial stem cells, allowing for long-term preservation of these cells without apparent harmful effects on their function (6).

Cryopreservation can reduce the level of spermatozoa antioxidants because of increasing the susceptibility of these cells to peroxidative damages after cryopreservation (9). In both in vivo and in vitro experiments, the melatonin was found to protect tissues against oxidative damages induced by numerous free radicals (10, 11).

The neurohormone melatonin is the major secretory product of the pineal gland. Some physiologic functions of melatonin are: circadian rhythm functions, sleeping and waking up, sexual activity, and reproductive functions, tumor growth, immune response, and aging (12). 

It can also act as a strong free radical scavenger and antioxidant (11). Beneficial effects of melatonin in protecting spermatozoa from different kinds of injuries have been reported in the literature. Some researchers showed the anti-apoptotic effects of melatonin on tissues (13, 14). Melatonin can apparently reduce the histological apoptosis, while inducing proliferation of the cells (15). Melatonin could improve cumulus-oocyte complexes quality in vitrified thawed media of murine ovaries (16). However, in another study melatonin did not reduce the injury induced by vitrified thawed media in immature testicular tissues (17).

To our knowledge, there was no any report in the literature regarding the condition of the cryopreserved testicular cells exposed to the freezing media containing melatonin. Therefore, this study determines whether the melatonin ameliorate the apoptosis rate of testicular cells after cryopreservation.

## Materials and methods

**Animals**

In this experimental study, inbred NMRI mice (7-10 days old males) were obtained from Physiology Research Center, Ahvaz Jundishapur University of Medical Sciences, experimental animal Research Center. Mice were euthanized by with ether and then testes were removed. Finally, animals were sacrificed after cervical dislocation. This study was approved by the Ethics Committee of Ahvaz University and carried out in an ethically way by following the provided guidelines (code; Ajums.rec.1392.125 date; 2013.10.5). 

**Cell isolation Procedure**

After removal of tunica albuginea, testes tissues were digested by two-step enzymatic procedure in Milazzo *et al* way by some modifications (18). Briefly, testicular cells were digested by incubation in 1 mg/ml collagenase type IV (Sigma, St. Louis, MO, USA) and 4 mg/ml DNase (Sigma, St. Louis, MO, USA) in 37^º^C incubator for 15 min while being pipetted several times.

Then, in the second step, after centrifuging of the suspension (300 g for 5 minutes), the supernatant was discarded and the cells were re-suspended in the media containing 0.25% trypsin/1 µm Ethylene Diamine Tetraacetic Acid (EDTA) (Invitrogen, Carlsbad, CA) and 0.06 mg/ml DNase I (Sigma, St. Louis, MO, USA) for 5 min in 37^º^C incubator. The enzymatic reaction was stopped by adding 10% fetal bovine serum (FBS). Then, the cells were filtered through 70 µm screens for producing a single cell suspension. Approximately, 4×10^5^ cells were collected from a neonatal testis by this procedure.

**Cryopreservation**

For freezing the cells, cryopreservation medium containing 10% (V/V) dimethyl sulfoxide (DMSO) (Sigma, St. Louis, MO, USA), 10% FBS (Gibco, USA), 30 mg/ml penicillin, 50 mg/ml streptomycin (Gibco, USA) was used. The freezing solution was diluted with Dulbecco's Modified Eagle's Medium (DMEM) (Sigma, St. Louis, MO, USA). In brief, after cell count and viability assessment, testicular cell suspensions were aliquoted in 0.5 ml volume (2×10^6^ cells per ml) in two separate cryotubes of 1.8 ml (Jet Biofil, CO, Ltd, China). 

Then, an equal volume of 2×concentrated freezing medium was added to the tube during 10-15 min. Cell suspensions were divided into two groups and 100µM melatonin (Sigma-Aldrich, USA) were added in one group. Dose selection was based on previous work (16). The viability of the cells was assessed again and after gently mixing by inverting the vials, samples was preserved in -70^º^C overnight. Then they were placed directly into the liquid nitrogen tank at -196^o^C for long term (20).

**Thawing procedure**

Cryoviales were held in a water bath (38^º^C) until defreeze, and then transferred into a tube and diluted slowly by adding two volumes, dropwise, of DMEM containing 10% FBS (19).

**Cytotoxicity assessment**

The lactate dehydrogenase (LDH) released from damaged plasma membranes into the extracellular fluid was determined using the commercially available Cytotoxicity Detection Kit (LDH) (Roche Diagnostics, Mannheim, Germany). The test was performed according to the manufacturer instructions and assessed by ELISA reader at 492 nm. High control determined the maximum releasable LDH activity in the cells. High control medium was supplied by addig the lysis buffer (Roche Diagnostics, Mannheim, Germany) in the medium. Each test was repeated in triplicate and measured by ELISA reader.

**Detection of DNA fragmentation**

Apoptotic cells incidence was quantified by flow cytometry using mouse monoclonal antibody, which detected the DNA fragmentation of apoptotic cells. As the DNA breaks, a large number of 3´ hydroxyl ends expose. For detection of apoptotic cells, the APO-Brdu TUNEL Assay Kit (Invitrogen, Carlsbad, CA) was used. The procedure was performed according to the kit instruction. Briefly, approximately 1×10^6^ cells were washed in phosphate-buffered saline (PBS) (Gibco, USA), fixed 15 minutes in 1% paraformaldehyde (Sigma-Aldrich, saint-Quentin fallavier, France) and 5 minutes in ethanol 70% on ice. Then, the cells were incubated in DNA labeling solution in a water bath at 37^o^C while being shaken for 15 minutes. 

Antibody staining solution (100 μL) containing 5μL of the Fluorescein Isothiocyanate (FITC) dye-labeled anti-BrdU mouse monoclonal antibody with 95 μL of rinse buffer were added to each sample and then they were incubated at the room temperature in the darkness. Moreover, the propidium iodide/RNase a staining buffer (0.5 mL) was added to samples and they were incubated at room temperature in the darkness again. At this stage, cell suspension was analyzed by a flow cytometer (Partec Flow Max, Germany). 

The positive and negative control cells were provided in the APO-BrdU™ TUNEL Assay Kit. A minimum of 10,000 cells were examined for each test. The fluorochrome was excited with 488 lasers. Green and red fluorescence were detected in the FL1 and FL3 channels of the flow cytometer, respectively. FL1 and FL2 fluorescence signals were recorded with logarithmic amplification and FL3 fluorescence signals were recorded with linear amplification. Apoptotic cells percentage were determined by dot plot of FL1 to FL3. All tests were run in duplicate.

**Statistical analysis**

All data were expressed as a mean±SD. Independent variables were compared using the independent-samples Student’s *t*-test and Statistical Package for the Social Sciences, version 16.0, SPSS Inc, Chicago, Illinois, USA (SPSS software). Results were considered statistically significant at p≤0.05.

## Results

**LDH test**

The cytotoxic effect of melatonin was evaluated via LDH. A summary of the mean levels of LDH is shown in table I. The basal level of LDH-release in the high control media was 1.16±0.63. The mean level of LDH leakage was increased in the melatonin supplemented media compared to the melatonin-free media (1.035±.079 vs. 0.755±0.183, respectively), but this increase was not significant (p≤0.05). The amount of melatonin cytotoxicity is 68% based on the kit manufactory formulation.

**Flow cytometry**

**Figure 1:**
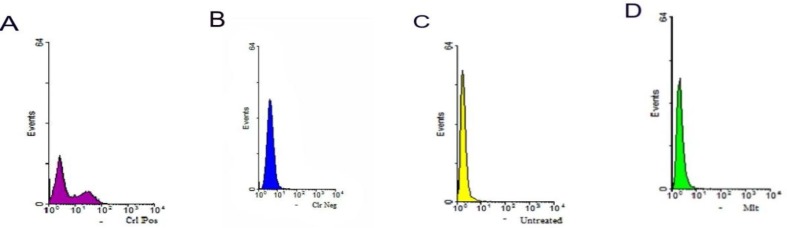
Flow cytometry study of neonate mouse testicular cells. Single parameter histograms are in FL1. The graphs display a single measurement parameter (relative fluorescence or light scatter intensity) on the x-axis and the number of event (cell count) on the y-axis. Melatonin increased the apoptotic cells which was kept in the cryopreserved media compared to the melatonin-free media (0.7±0.14 vs 0.23 ±0.03, respectively) (p=0.006). (A) Positive control. (B) Negative control. (C) Melatonin free group. (D) Melatonin group

**Figure 2:**
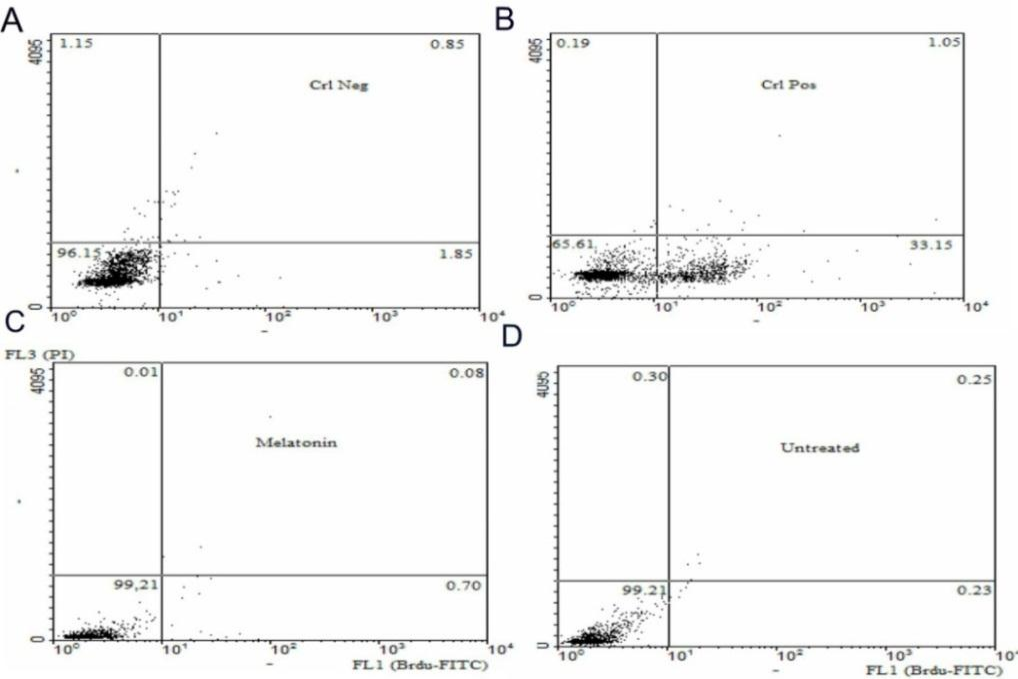
Dual-color dot plot of Brdu-FITC / Propidium Idide (PI) flow cytometry study. Cells were analyzed for Brdu-FITC in forward scatter and red fluorescence (PI) in side scatter. Lower left of each quadrant indicates cells not to stain (viability percentage) and lower right stained positive for Brdu- FITC showing apoptosis percent and upper right stained positive for Brdu- FITC and PI indicates the percent of late apoptosis and necrosis and upper left that stained with PI shows necrotic cells. The control groups were supplied with the kit. As it was showed, melatonin could significantly increase the apoptotic cells in the cryopreserved media (0.7±0.14 vs. 0.23±0.03, respectively) (p=0.006). (A) Negative control. (B) Positive control. (C) Melatonin group. (D) Melatonin free group

Histogram results of FL1 channel are illustrated in figure 1 and dot plot results are shown in figure 2. First, the flow cytometer was set by positive and negative controls. The level of apoptotic cells percentage in the media supplemented with melatonin was significantly increased in comparison to the melatonin-free media (0.23±0.03 vs 0.7±0.14, respectively) (p=0.006). However, there were no significant differences in viability and necrosis rates between these two groups (p≤0.05).

**Table I:** Toxicity assay data

**Variable**	**Repeat**			**Mean±SD**
	**1**	**2**	**3**	
Media containing melatonin	0.943	1.095	1.069	1.035±.079
Media without melatonin	0.968	0.623	0.676	0.755±0.183
High control media (Positive control)	1.115	1.146	1.238	1.166±0.63

Cytotoxicity test indicated that melatonin elevates LDH in cryopreserved testicular cells. This increase was not significant (p≤ 0.05).

## Discussion

In the present study, the effect of supplementation of the cryopreservation medium with melatonin on frozen testicular cells of the mouse was assessed. Melatonin which is the chief secretary product of the pineal gland has a very strong antioxidant activity, depending mainly on its capacity to act as an electron donor (11). Melatonin acts directly as a free radical scavenger, whereas the indirect actions of melatonin occur when it stimulates the endogenous antioxidant enzymes (20, 21). In vivo and in vitro, melatonin has been found to protect tissues against oxidative damages generated by a variety of toxic agents and metabolic processes, including oxidative damage in testis (22), chemotherapy-induced toxicity (23) and ischemia-reperfusion injuries in stomach, liver, and brain (24). However, in this study melatonin could not only prevent toxic effects of freezing on testicular cells, it also had toxic effects. Indeed, melatonin cannot prevent the release of LDH. LDH is a stable cytoplasmic enzyme presented in all cells. It is rapidly released into the cell culture supernatant upon damage of the plasma membrane (25, 26).

Also, in the current study, it is shown that supplementation of the cryopreserved medium with melatonin causes increased apoptotic cell death in testicular cells. Hemadi *et al* suggested that the use of melatonin right after thawing vitrified testis tissue and subsequent melatonin treatment during transplantation is a feasible approach to better maintain the potential of spermatogenesis kinetics, and it may serve as a promising method for graft preservation (16). These results are in contradiction with the current results. The differences may be due to the addition of melatonin to thawing solution by Hemadi *et al* whereas we added the melatonin to the cryopreservation media. Moreover, they freezed the whole testis, while we preserved testicular cells (15). Previously, a comparative study was performed in which cryopreservation of testicular tissue was carried out with vitrification medium supplemented with melatonin versus the melatonin-free medium. The increase in apoptosis was observed in the media supplemented with melatonin (17). These results are in agreement with our experiments. Mohamad-Ghasemi *et al* stated that melatonin has a protective effect on epididymal sperm parameters, seminal vesicle and epididymis morphology in mouse under treatment with chemotherapy (27). 

They declared that although the mechanism is not clear, it acts probably by decreasing oxidative stresses. In another study, it was achieved the protective effect of melatonin in busulfan treated mice (28). Apparently, melatonin treatment produces antioxidant effect in vivo. Succu *et al* claimed that adding melatonin to the ram semen vitrification solution protects the spermatozoa during cryopreservation in a dose-dependent manner. So, the difference in the melatonin dosage may cause to such different results (29). In another study, it was found that melatonin has an anti-apoptotic effects on germ cells (unpublished data). In that study, we purified the CD 90.1+germ cells by magnetic activated cell sorting (MACS). It appears that melatonin has an anti-oxidant effect on purified spermatogonial stem cell, but it has a contentious effect on gross population of testicular cells, so the results may be different if it is repeated by a pure population of spermatogonial stem cells. It can largely be provided by MACS method. The difference between actual absorption rate of melatonin with antioxidant effect and toxic effect may be another reason for these different results (29). Evaluation of data from prolonged multicentral studies seems to be essential for determination of the safe and effective dose of melatonin along with achieving maximum benefit and minimum adverse events of it (30).

## Conclusion

In conclusion, our results indicated that melatonin has apoptotic effect on testicular cells in cryopreservation media.

## Note

***This article extracted from Ph.D. thesis. (Mehri Mirhoseini)***
